# Identification and Characterization of Resistance Loci to Wheat Leaf Rust and Stripe Rust in Afghan Landrace “KU3067”

**DOI:** 10.3389/fpls.2022.894528

**Published:** 2022-06-28

**Authors:** Peipei Zhang, Caixia Lan, Ravi P. Singh, Julio Huerta-Espino, Zaifeng Li, Evans Lagudah, Sridhar Bhavani

**Affiliations:** ^1^State Key Laboratory of North China Crop Improvement and Regulation, College of Plant Protection, Hebei Agricultural University, Baoding, China; ^2^Hubei Hongshan Laboratory, College of Plant Science & Technology, Huazhong Agricultural University, Wuhan, China; ^3^International Maize and Wheat Improvement Center (CIMMYT), Texcoco, Mexico; ^4^Campo Experimental Valle de México the National Institute of Forestry, Agricultural and Livestock Research (INIFAP), Texcoco, Mexico; ^5^Commonwealth Scientific and Industrial Research Organisation (CSIRO) Plant Industry, Canberra, ACT, Australia

**Keywords:** genetic analysis, molecular mapping, wheat rusts, APR genes, landraces

## Abstract

Leaf rust and stripe rust are important wheat diseases worldwide causing significant losses where susceptible varieties are grown. Resistant cultivars offer long-term control and reduce the use of hazardous chemicals, which can be detrimental to both human health and the environment. Land races have been a valuable resource for mining new genes for various abiotic and biotic stresses including wheat rusts. Afghan wheat landrace “KU3067” displayed high seedling infection type (IT) for leaf rust and low IT for stripe rust; however, it displayed high levels of field resistance for both rusts when tested for multiple seasons against the Mexican rust isolates. This study focused on identifying loci-conferring seedling resistance to stripe rust, and also loci-conferring adult plant resistance (APR) against the Mexican races of leaf rust and stripe rust. A backcrossed inbred line (BIL) population advanced to the BC1F_5_ generation derived from the cross of KU3067 and Apav (triple rust susceptible line) was used for both, inheritance and QTL mapping studies. The population and parents were genotyped with Diversity Arrays Technology-genotyping-by-sequencing (DArT-Seq) and phenotyped for leaf rust and stripe rust response at both seedling and adult plant stages during multiple seasons in Mexico with relevant pathotypes. Mapping results identified an all-stage resistance gene for stripe rust, temporarily designated as *YrKU*, on chromosome 7BL. In total, six QTL-conferring APR to leaf rust on 1AS, 2AL, 4DL, 6BL, 7AL, and 7BL, and four QTL for stripe rust resistance on 1BS, 2AL, 4DL, and 7BL were detected in the analyses. Among these, pleiotropic gene *Lr67*/*Yr46* on 4DL with a significantly large effect is the first report in an Afghan landrace-conferring resistance to both leaf and stripe rusts. *QLr.cim-7BL*/*YrKU* showed pleiotropic resistance to both rusts and explained 7.5–17.2 and 12.6–19.3% of the phenotypic variance for leaf and stripe rusts, respectively. *QYr.cim-1BS* and *QYr.cim-2AL* detected in all stripe environments with phenotypic variance explained (PVE) 12.9–20.5 and 5.4–12.5%, and *QLr.cim-6BL* are likely to be new. These QTL and their closely linked markers will be useful for fine mapping and marker-assisted selection (MAS) in breeding for durable resistance to multiple rust diseases.

## Introduction

Wheat leaf rust and stripe rust caused by obligate biotrophic fungus *Puccinia triticina* (*Pt*) and *P*. *striiformis* f. sp. *tritici* (*Pst*), respectively, are the most important foliar diseases of wheat worldwide. Leaf rust is the most commonly occurring disease and can cause yield losses up to 40% under favorable conditions (Knott, 1989). Stripe rust occurs in cool temperate regions and can cause yield losses ranging from 10 to 70% and up to 100% in highly susceptible cultivars (Chen, 2005). Although fungicides can effectively control wheat rusts, growing resistant cultivars is a more efficient, economic, environment-friendly, and long-term strategy to minimize losses.

Resistance to wheat rusts can be broadly classified either as race-specific or as race non-specific resistances (Johnson, 1988). Race-specific resistance (or) seedling resistance (or) all-stage resistance is often characterized by a strong to moderate immune response usually associated with the hypersensitive response that fully curtails fungal infection and sporulation at all developmental stages if the pathogen possesses a corresponding avirulence gene (Flor, 1942). These resistance genes are usually effective against a single race or a few races of the pathogen. This kind of resistance may lose effectiveness when new virulent pathotypes arise through mechanisms of mutation or recombination. Race non-specific resistance on the other hand is under polygenic control and usually expressed susceptible response at the seedling stage and expressed quantitatively at post-seedling growth stages either as slow rusting or partial resistance, or adult plant resistance (APR). It is usually characterized by lower frequencies of infection, longer latency period, smaller uredinium, and less urediniospore production (Caldwell, 1968). The phenotypic effects of such genes are usually minor; however, several of such genes with additive effects can be combined leading to near levels of immunity (Singh et al., 2012). These genes usually are race non-specific and are effective against multiple races of the pathogen and compared with race-specific resistance, therefore, conditioning broader effectiveness and enhanced durability.

To date, 80 leaf rust resistance genes and 83 stripe rust resistance genes are officially cataloged in wheat (Li et al., 2020; Kumar et al., 2021) and most of the identified genes showed race-specific resistance. Only a few genes, for example, *Lr34*/*Yr18*/*Pm38*/*Sr57* (Singh et al., 2012), *Lr46*/*Yr29*/*Pm39*/*Sr58* (Singh et al., 2013), *Lr67*/*Yr46*/*Pm46*/*Sr55* (Herrera-Foessel et al., 2011), and *Lr68* (Herrera-Foessel et al., 2012) are also known to confer pleiotropic effect on the resistance. In addition to the formally named genes, 249 leaf rust and 327 stripe rust resistance QTL have been reported on every chromosome (Wang and Chen, 2017; Pinto da Silva et al., 2018). Even though multiple genes have been characterized and cataloged only a handful of genes, conferring adequate levels of resistance to prevalent rust races have made an impact in rust resistance breeding as the majority of them are effective to specific races (or) do not provide adequate levels of resistance (or) are associated with negative linkage drag with yield and other traits (Bhavani et al., 2019). The advent of molecular markers and the availability of reference maps and completely annotated wheat genome has greatly facilitated rapid gene discovery and characterization (Clavijo et al., 2017; Rosewarne et al., 2013).

Molecular markers have been widely used for mapping APR genes for rust resistance through QTL analysis. Several high-throughput genotyping technologies, such as single nucleotide polymorphism (SNP) and genotyping-by-sequencing (GBS) have greatly facilitated the identification and characterization of genomic regions of complex traits such as rust resistance (Bhavani et al., 2021). Diversity arrays technology sequencing (DArT-Seq) developed by the Diversity Arrays Technology Pty Ltd (Canberra, Australia) is a new approach based on traditional DArT complexity reduction methods and Next Generation Sequencing (NGS) techniques (http://www.diversityarrays.com/dart-application-dartseq).

Diversity arrays technology sequencing offers affordable genome profiling through the generation of high-density SNPs as well as PAV (presence/absence variation) markers. This technology can be better used in linkage map construction and accelerates high-resolution mapping and detailed genetic dissection of traits (Raman et al., 2014) and has been extensively used to study the diversity of wheat accessions in the CIMMYT gene bank (Sansaloni et al., 2020).

The wheat line “KU3067,” a landrace from Afghanistan, was stored in the National Bio-Resource Project of Japan in 1956 (Tanaka et al., 2008). It displays high levels of resistance to leaf rust and stripe rust in the Mexico's field conditions. This study aimed to determine the genetic basis of leaf rust, stripe rust resistance in KU3067, and identify molecular markers linked to these QTL that can be used in breeding.

## Materials and Methods

### Plant Materials and Pathotypes

The mapping population comprised of 148 BC1F_5_ lines (backcross-inbred lines, BILs) was derived from the cross of KU3067 and Apav (Apav was used as the recurrent susceptible parent). KU3067 showed a high level of APR to both rusts in field trials, whereas Apav derived from the “Avocet-*YrA*/Pavon 76” mapping population was completely susceptible to the three rusts. Predominant Mexican *Pst* pathotype MEX14.191 and two *Pt* pathotypes (MBJ/SP and MCJ/SP) were used to test the BILs in the greenhouse and field. These *Pt* and *Pst* pathotypes were available at the greenhouse at the International Maize and Wheat Improvement Center (CIMMYT).

### Evaluation of Seedling Responses to Leaf and Stripe Rust in the Greenhouse

Seedling evaluations of KU3067, Apav and the BIL population were conducted in the greenhouse using *Pst* pathotype Mex14.191 (avirulence/virulence: *Yr1, 4, 5a, 10, 15, 24, 26, 5b, Poll*/*Yr2, 3, 6, 7, 8, 9, 17, 27, 31, A*) (Randhawa et al., 2018), and *Pt* pathotypes MBJ/SP [avirulence/virulence: *Lr2a, 2b, 2c, 3ka, 9, 16, 19, 21, 24, 25, 28, 29, 30, 32, 33, 36*/*1, 3, 3bg, 10, 11, 12, 13, 14a, 14b, 15, 17a, 18, 20, 23*, (*26*), *27*+*31*] and MCJ/SP (additional virulence to *Lr16*) [avirulence/virulence: *Lr2a, 2b, 2c, 3ka, 9, 19, 21, 24, 25, 28, 29, 30, 32, 33, 36*/*1, 3, 3bg, 10, 11, 12, 13, 14a, 14b, 15, 16*,*17a, 18, 20, 23*, (*26*), *27*+*31*] (Huerta-Espino et al., 2020). In total, thirty differential lines with known stripe rust resistance genes (mostly in the Avocet background) were also included in the seedling experiment, and a set of forty-eight lines with known LR genes, mostly in “Thatcher” background, were also included for leaf rust evaluations. Seedlings were inoculated at the two-leaf stage by spraying urediniospores suspended in the lightweight mineral oil Soltrol-170 (Chempoint.com) at a concentration of 2–3 mg/ml using an atomizer. Inoculated plants were placed in a dew chamber at 7°C for 18 h, and then transferred to the greenhouse maintained at 15–18°C. Infection types (ITs) were recorded 12–14 days after inoculation. Leaf rust was evaluated according to the Stakman 0 to 4 scale as modified by Roelfs et al. (1992); for stripe rust, ITs were recorded according to the 0 to 9 scale as modified by McNeal et al. (1971). BILs with ITs of 0–4, 5–6, and 7–9 to *Pst* are categorized as resistant, intermediate, and susceptible, respectively.

### Field Experiments

Leaf rust field evaluations were carried out in CIMMYT (International Maize and Wheat Improvement Center) research stations in Mexico at El Batan (19.5277° N, 98.8569° W, and 2,249 masl) during the 2016 and 2017 cropping seasons where leaf rust screening can be carried out successfully (hereafter referred to as LR16B and LR17B, respectively), and at CENEB-Campo Experimental Norman E. Borlaug (27.3710° N, 109.9305° W, and 39 masl) located in Ciudad Obregon during the 2016–2017 and 2017–2018 growing seasons which is also the favorable environment for leaf rust (LR17O and LR18O). Stripe rust trials were conducted at Toluca which is a disease phenotyping platform for yellow rust and Septoria (19.5562° N, 99.2675° W, and 2,640 masl) Mexico, during the 2017 and 2018 crop seasons (YR17, YR18 experiments). Field plots consisted of 0.7-m paired rows with approximately 60 plants on each line. Avocet near-isolines (NILs) carrying *Yr24* and *Yr26* were used as spreaders in leaf rust studies, whereas a mixture of Morocco, Avocet NIL carrying *Yr31*, and six lines possessing the *Yr27* gene, derived from the cross Avocet/Attila, were used as stripe rust spreaders. The spreader cultivars were planted as hills in the middle of a 0.3-m pathway on one side of each plot and planted perpendicular and adjacent to the test rows. Artificial inoculations on spreader rows and hills were carried out twice at weekly intervals by spraying aqueous suspensions of urediniospores of equal mixtures of *Pt* pathotypes MBJ/SP and MCJ/SP for leaf rust and Mex14.191 for stripe rust which were suspended in Soltrol 170 at a concentration of 2–3 mg/ml and dispensed onto the spreader rows at the tillering stage (Feekes growth stage 5; Large, 1954). The avirulence/virulence formulas of MBJ/SP and MCJ/SP were described in Herrera-Foessel et al. (2012) and of race Mex14.191 in Zhang et al. (2019). The host response to infection in adult plants was determined according to Roelfs et al. (1992). Disease severities were scored following the 0–100% visual ratings two or three times at weekly intervals according to the Modified Cobb Scale (Peterson et al., 1948). The area under the disease progress curve (AUDPC) was calculated using the method suggested by Bjarko and Line (1988).


$$\begin{array}{l} \text{AUDPC}\;=\;\sum_{i=1}^{n}[({\text{X}}_{i\;+\;1}\;+\;{\text{X}}_{i})/2][{\text{T}}_{i\;+\;1}\;-{\text{T}}_{i}] \end{array}$$

where X_*i*_ is the disease severity on assessment date *i*, T_*i*_ is the number of days after inoculation on assessment date *i*, and n is the number of disease assessments. Maximum disease severity (MDS, %) data and AUDPC were used for QTL analysis.

### Genetic and Statistical Analyses

Correlation analysis between MDS in different environments was conducted using bivariate two-tailed Pearson's correlation coefficients by IBM SPSS Statistics 21.0 (IBM Corp., Armonk, NY). The number of genes were estimated using chi-squared tests using the expected and observed segregation ratios. Based on disease severity and infection response, BILs were broadly classified into three phenotypic categories–homozygous parental type resistant (HPTR), homozygous parental type susceptible (HPTS), and intermediate types (others)–following Singh and Rajaram (1992). Chi-squared tests were carried out to test the best fit for different gene segregation ratios.

### Linkage Map Construction and QTL Detection

Genomic DNA of the parents and BILs were isolated from non-infected tissues by the CTAB method (Sharp et al., 1988). DNA concentration was measured using Thermo Scientific NanoDrop 8000. The 148 BILs and parents were genotyped with DarT-Seq in CIMMYT's Biotech Laboratory. The linkage map was constructed using the MAP function in IciMapping 4.1 (http://www.isbreeding.net/software/?type=detail&id=18, Li et al., 2007). QTL analysis was conducted with the ICIM–ADD function in BIP using the software QTL IciMapping 4.1 through 1,000 permutations at *P* = 0.01 (Li et al., 2007). Stepwise regression analysis was used to detect the percentages of phenotypic variance explained (PVE, *R*^2^) by individual QTL and additive effects at the LOD peaks. The linkage map was drawn using MapChart 2.3 (http://www.earthatlas.mapchart.com/, Voorrips, 2002). The sequences of all the markers were subjected to the BLAST against the Chinese Spring reference sequence (version 2.0 https://urgi.versailles.inra.fr/blast_iwgsc/blast.php, IWGSC, 2018) in order to determine physical positions.

Phenotypic distributions of stripe rust and leaf rust MDS were also compared between two groups of BILs that were classified based on the presence or absence of a QTL. Finally, all the BILs were grouped into different QTL genotypic classes by the flanking markers to check the additive effect, and disease severity was calculated by averaging rust scores within a QTL group across environments.

## Results

### Seedling Response to Leaf Rust and Stripe Rust

In the seedling test, KU3067 and Apav displayed susceptible IT (3+ and 4) against both *Pt* races MBJ/SP and MCJ/SP based on a 0–4 scale. For the stripe rust, KU3067 expressed resistant IT 3 when tested with *Pst* Mex14.191, whereas Apav produced susceptible IT 7/8 based on the 0–9 scale. When tested on 148 BIL lines, 42 lines were found resistant, 101 lines were susceptible, and 6 lines showed segregation. Chi-squared analysis conformed to the expected frequency for a single stripe rust resistance gene in the seedling test against *Pst* Mex14.191 (Table 1). The resistance gene was temporarily designated as *YrKU*.

**Table 1:** Estimated number of resistance genes that confer seedling resistance to stripe rust and adult plant resistance to leaf rust and stripe rust in 148 KU3067 × Apav BILs based on Mendelian segregation analysis.

**Response category of BILs**	**No. of BILs (APR)** ^a^						**No. of BILs (seedlings)^b^**
	**LR16B**	**LR17B**	**LR17O**	**LR18O**	**YR17**	**YR18**	**Mex14.191**
HPTS^c^	34	35	63	68	42	30	101
HPTR^d^	2	1	3	1	1	1	42
OTHER^e^	111	112	82	79	104	117	6
Missing	1	0	0	0	1	0	0
Total	148	148	148	148	148	148	148
No. of genes	4	4	3	3	4	4	1
*P* Value^f^	0.02	0.26	0.52	0.25	0.7	0.05	0.29

a *Disease severity and host response to infection determined for leaf rust at El Batán 2016 (LR16B) and 2017 (LR17B); Ciudad Obregón during the 2016–2017 (LR17O) and 2017–2018 (LR18O) seasons, and for stripe rust at Toluca during the 2017 (YR17) and 2017 (YR18) seasons*.

b *Seedling tests with Pst pathotype Mex14.191 conducted twice in the greenhouse to determine the seedling response category of BILs*.

c *Homozygous parental type susceptible*.

d *Homozygous parental type resistant*.

e *Lines with responses different from the two parents*.

f *P-value is for the χ^2^ test. The expected ratio of BILs grouped under HPTS, HPTR, and OTHER are 0.734:0.234:0.0313, 0.396:0.0129:0.591, and 0.291:0.003:0.706 for segregation of 1, 3, and 4 independently inherited genes, respectively, in the F_5_-derived F_6_ generation*.

### Characterization of Leaf Rust and Stripe Rust Resistance in the Field

**Figure 1:**
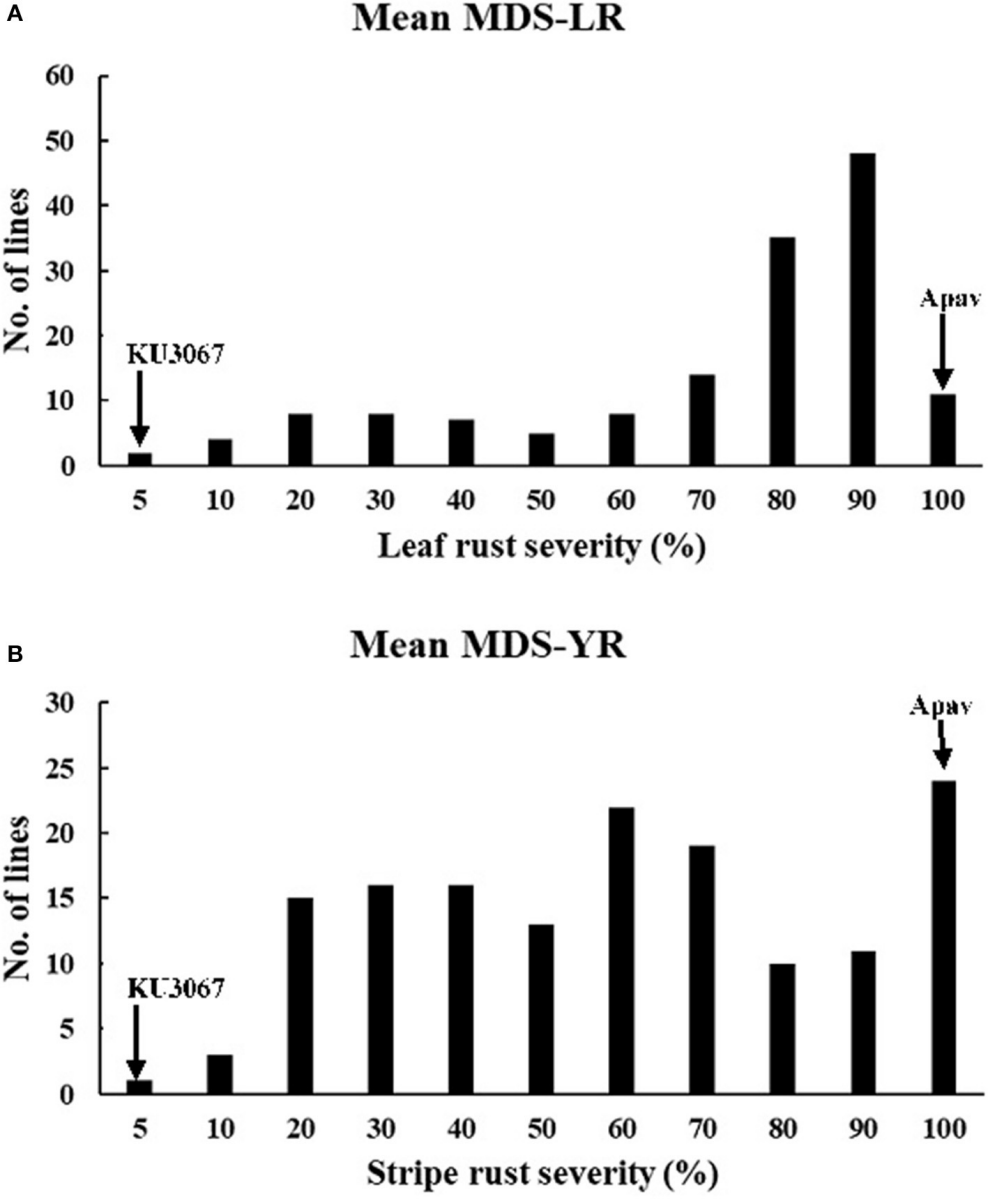
Frequency distributions of KU3067/Apav BILs lines for mean LR **(A)** and YR **(B)** MDS in the field conditions. Mean values for the parents, KU3067 and Apav, are indicated by arrows.

In the field trials, the MDS and IT reaction to leaf rust was 1MSS for KU3067 and 100S for Apav across all the seasons. Mean leaf rust severities on BILs ranged from 50.1 to 79.0% during all the leaf rust trials (Table 2). The frequency distribution of BILs for leaf rust severity was continuous over the four environments (Figure 1A), indicating the polygenic inheritance of APR to leaf rust in the population. Genetic analyses by Mendelian segregation analysis (Table 1) indicated the presence of 3–4 APR genes that confer resistance to the leaf rust in the population.

**Table 2:** Summary of MDS in the KU3067 × Apav BIL population phenotyped for leaf rust and stripe rust.

**Parent/Parameter**	**LR16B**	**LR17B**	**LR17O**	**LR18O**	**LRM**	**YR17**	**YR18**	**YRM**
APAV	100S	100S	100S	100S	100S	100S	100S	100S
KU3067	1MSS	1MSS	1MSS	1MSS	1MSS	1R	1R	1R
Population mean	50.1	67.2	72.5	79.0	67.2	67.8	60.7	64.3
Range	1–100	1–100	1–100	5–100	3–100	1–100	5–100	3–100

KU3067 and Apav displayed MDS and IT for stripe rust of 5R and 100S under field conditions. The mean MDS for all the BILs was 67.8% and 67.0% in YR2017 and YR2018, respectively. The stripe rust MDS scores for the 148 BILs across all environments also showed continuous distributions (Figure 1B). Four genes were estimated to provide resistance to stripe rust using the Mendelian segregation analysis (Table 1).

MDS scores for leaf rust across all the crop seasons were significantly correlated with correlation coefficients ranging from 0.73 to 0.88 (*P* < 0.001; Table 3). For stripe rust, the correlation coefficient for YR2017 and YR2018 was 0.84 (*P* < 0.001). The coefficients of the correlation between stripe rust and leaf rust disease scores ranged from 0.61 to 0.76 across experiments, indicating the presence of pleiotropic genes conferring resistance to both the rusts.

**Table 3:** Pearson correlation coefficients (r) for two-way comparisons of leaf rust and stripe rust severity data from different environments.

**Environment**	**LR16B**	**LR17B**	**LR17O**	**LR18O**	**YR17**
LR17B	0.73^**^				
LR17O	0.75^**^	0.76^**^			
LR18O	0.79^**^	0.77^**^	0.88^**^		
YR17	0.69^**^	0.76^**^	0.74^**^	0.73^**^	
YR18	0.61^**^	0.73^**^	0.65^**^	0.65^**^	0.84^**^

** *P < 0.01*.

### Linkage Map Construction and Mapping the Seedling Stripe Rust Resistance Gene *YrKU*

**Figure 2:**
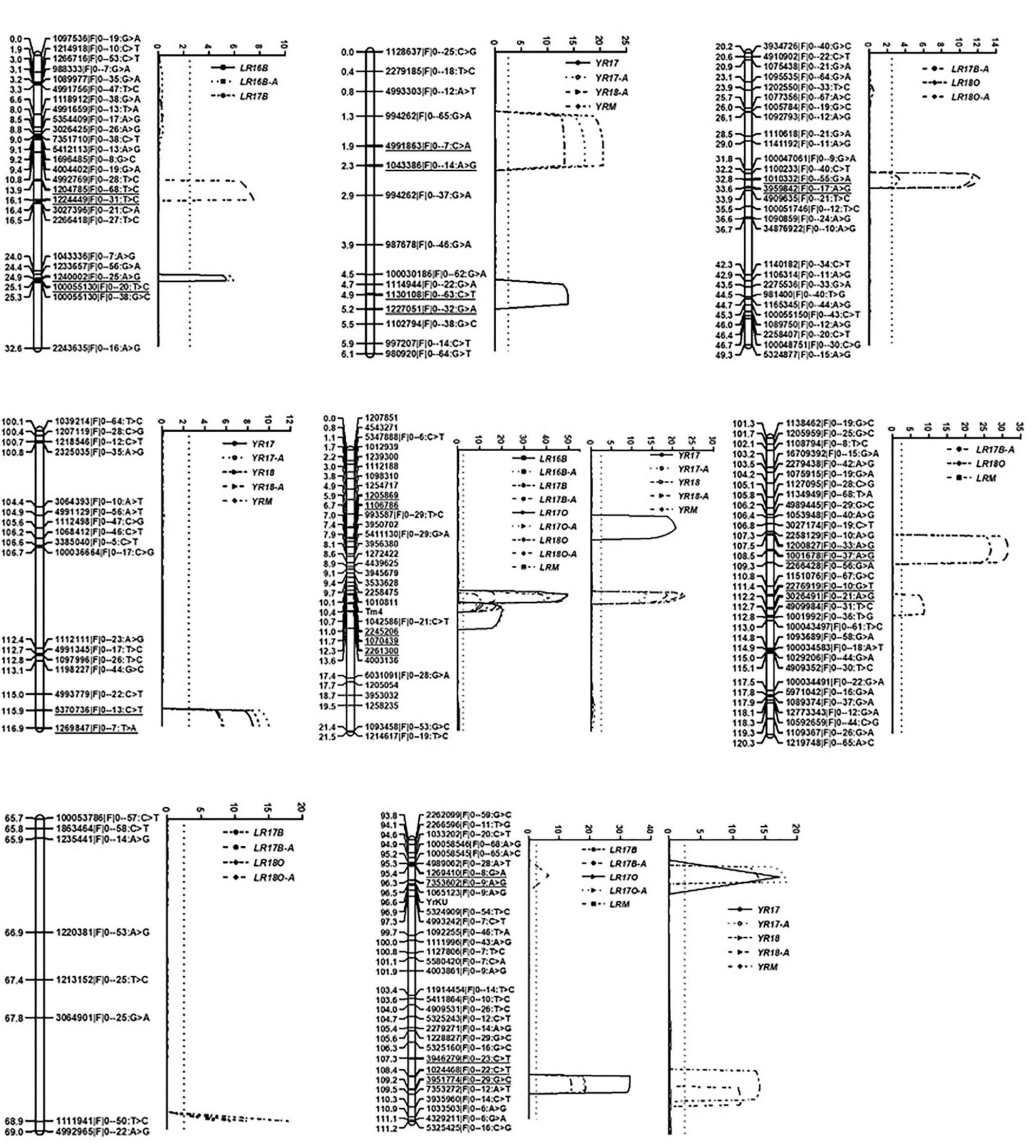
Likelihood plots of QTL for APR to leaf rust and stripe rust identified by IciMapping V4.1 in the KU3067 × Apav BILs population. The significant LOD threshold was detected based on 1,000 permutations. Positions (in cM) of cumulated genetic distances of linkage group along chromosomes are shown on the left axes and for molecular markers on the right. The marker interval of the QTL was underlined.

A total of 4,053 markers were used to construct the linkage map. The resulting linkage map comprised 40 linkage groups and a total map distance of 2,863.9 cM with A 1,190.5 cM, B 930.7 cM and D 742.7 cM, respectively (Supplementary Table 1). *YrKU* was mapped on 7BL and flanked by SNP markers 1070196|F|0–47:A>G and 5324909|F|0–54:T>C with genetic resistance 0.02 and 0.33 cM, respectively (Figure 2).

### QTL Mapping for Leaf Rust and Stripe Rust Resistance

#### QTL Mapping of APR to Leaf Rust

Six leaf rust APR QTL were identified on chromosomes 1AS, 2AL, 4DL, 6BL, 7AL, and 7BL, and designated as *QLr.cim-1AS, QLr.cim-2AL, QLr.cim-4DL, QLr.cim-6BL, QLr.cim-7AL*, and *QLr.cim-7BL*, respectively (Table 4; Figure 2). All the resistance alleles were from KU3067. *QLr.cim-1AS* was detected in LR16B, LR16B-A (AUDPC for leaf rust at El Batán in 2016) and LR17B and explained 9.4–12.0% of the variation. *QLr.cim-2AL* with relatively smaller effects on disease response was identified in LR17B-A, LR18O, and LR18O-A with PVE ranging from 3.0 to 11.4%. *QLr.cim-4DL* with large effects was stably detected in all the environments and explained 10.9–56.4% of the phenotypic variances for leaf rust. *QLr.cim-6BL* was detected in LR17B-A, LR18O, and LRM (mean MDS across all the leaf rust environments) and explained 8.7–17.9% of the phenotypic variance. *QLr.cim-7AL* in the marker interval 4992965|F|0–22:A>G and 1111941|F|0–50:T>C was identified in LR17B, LR17B-A, LR18O, and LR18O-A with PVE ranging from 6.7–21.7%. *QLr.cim-7BL* was detected in LR17B, LR17B-A, LR17O, LR17O-A, and LRM and explained 7.5–17.2% of the phenotypic variance.

**Table 4:** Quantitative trait locus/loci for MDS to leaf rust and stripe rust by ICIM in the BIL population from Ku3067/Apav.

**QTL^a^**	**Environment^b^**	**Position (cM|)^c^**	**Marker interval**	**Physical position (Mb)**	**LOD^d^**	**PVE(%)^e^**	**ADD^f^**
*QLr.cim-1AS*	LR16B	25.0	*1240002|F|0–25:A>G−100055130|F|0–20:T>C*	1.1–46.1	5.3	9.4	−7.4
	LR16B-A	25.0	*1240002|F|0–25:A>G−100055130|F|0–20:T>C*	1.1–46.1	5.9	9.6	−34.8
	LR17B	16.0	*1204785|F|0–68:T>C−1224449|F|0–31:T>C*	4.6–8.0	7.5	12.0	−8.6
*QYr.cim-1BS*	YR17	5.0	*1130108|F|0–63:C>T−1227051|F|0–32:G>A*	3.7–31.9	13.9	12.9	−7.9
	YR17-A	2.0	*4991863|F|0–7:C>A−1043386|F|0–14:A>G*	3.7–7.0	17.1	15.5	−108.9
	YR18-A	2.0	*4991863|F|0–7:C>A−1043386|F|0–14:A>G*	3.7–7.0	13.2	14.1	−55.2
	YRM	2.0	*4991863|F|0–7:C>A−1043386|F|0–14:A>G*	3.7–7.0	20.6	20.5	−10.6
*QLr.cim-2AL*	LR17B-A	32.0	*1010332|F|0–55:G>A−3959842|F|0–17:A>G*	644.4–654.3	3.3	3.0	−23.0
	LR18O	32.0	*1010332|F|0–55:G>A−3959842|F|0–17:A>G*	644.4–654.3	12.1	11.4	−12.0
	LR18O-A	32.0	*1010332|F|0–55:G>A−3959842|F|0–17:A>G*	644.4–654.3	11.2	7.8	−46.8
*QYr.cim-2AL*	YR17	116.0	*5370736|F|0–13:C>T−1269847|F|0–7:T>A*	762.6–764.7	8.5	12.5	−9.1
	YR17-A	116.0	*5370736|F|0–13:C>T−1269847|F|0–7:T>A*	762.6–764.7	10.0	8.5	−40.0
	YR18-A	116.0	*5370736|F|0–13:C>T−1269847|F|0–7:T>A*	762.6–764.7	5.6	7.0	−4.9
	YR18-A	116.0	*5370736|F|0–13:C>T−1269847|F|0–7:T>A*	762.6–764.7	5.8	5.4	−32.5
	YRM	116.0	*5370736|F|0–13:C>T−1269847|F|0–7:T>A*	762.6–764.7	9.1	7.4	−6.0
*Lr67*	LR16B	12.2	*1070439–2261300*	234.4–434.2	20.3	47.2	−16.1
	LR16B-A	12.2	*1070439–2261300*	234.4–434.2	20.9	45.5	−73.7
	LR17B	11.2	*2245206–1070439*	234.4–419.3	17.4	33.6	−15.7
	LR17B-A	11.2	*2245206–1070439*	234.4–419.3	9.5	10.9	−37.5
	LR17O	11.2	*2245206–1070439*	234.4–419.3	48.9	41.1	−31.4
	LR17O-A	11.2	*2245206–1070439*	234.4–419.3	41.9	54.5	−183.5
	LR18O	11.2	*2245206–1070439*	234.4–419.3	49.4	48.1	−27.0
	LR18O-A	11.2	*2245206–1070439*	234.4–419.3	40.2	56.4	−111.5
	LRM	11.2	*2245206–1070439*	234.4–419.3	42.8	50.0	−20.9
*Yr46*	YR17	6.2	*1205869–1106786*	63.5–67.5	19.7	21.7	−11.4
	YR17-A	11.2	*2245206–1070439*	234.4–419.3	13.2	12.1	−112.2
	YR18	11.2	*2245206–1070439*	234.4–419.3	18.0	29.6	−12.2
	YR18-A	11.2	*2245206–1070439*	234.4–419.3	22.2	29.8	−92.2
	YRM	11.2	*2245206–1070439*	234.4–419.3	20.9	22.8	−13.0
*QLr.cim-6BL*	LR17B-A	112.0	*2276919|F|0–10:G>T−3026491|F|0–21:A>G*	530.1–585.2	8.5	8.7	−30.3
	LR18O	108.0	*1200827|F|0–33:A>G−1001678|F|0–37:A>G*	495.2–576.7	31.2	17.7	−14.5
	LRM	108.0	*1200827|F|0–33:A>G−1001678|F|0–37:A>G*	495.2–576.7	26.4	17.9	−11.1
*QLr.cim-7AL*	LR17B	68.0	*1111941|F|0–50:T>C−4992965|F|0–22:A>G*	699.0–701.5	9.9	17.3	−8.9
	LR17B-A	68.0	*1111941|F|0–50:T>C−4992965|F|0–22:A>G*	699.0–701.5	18.4	21.7	−52.7
	LR18O	68.0	*1111941|F|0–50:T>C−4992965|F|0–22:A>G*	699.0–701.5	7.8	6.7	−8.0
	LR18O-A	68.0	*1111941|F|0–50:T>C−4992965|F|0–22:A>G*	699.0–701.5	12.0	8.4	−41.6
*QLr.cim-7BL*	LR17B	96.0	*1269410|F|0–8:G>A−7353602|F|0–9:A>G*	728.9–729.8	6.6	11.9	−6.4
	LR17B-A	109.0	*1024468|F|0–22:C>T−3951774|F|0–29:G>C*	752.4–754.2	18.7	17.2	−11.7
	LR17O	109.0	*1024468|F|0–22:C>T−3951774|F|0–29:G>C*	752.4–754.2	32.9	16.3	−17.6
	LR17O-A	109.0	*1024468|F|0–22:C>T−3951774|F|0–29:G>C*	752.4–754.2	18.0	12.5	−77.5
	LRM	109.0	*1024468|F|0–22:C>T−3951774|F|0–29:G>C*	752.4–754.2	14.1	7.5	−7.2
*QYr.cim-7BL*	YR17	96.0	*1269410|F|0–8:G>A−7353602|F|0–9:A>G*	728.9–729.8	17.2	19.3	−12.8
	YR17-A	96.0	*1269410|F|0–8:G>A−7353602|F|0–9:A>G*	728.9–729.8	18.1	17.1	−117.9
	YR18	109.0	*1024468|F|0–22:C>T−3951774|F|0–29:G>C*	752.4–754.2	11.1	15.1	−7.7
	YR18-A	108.0	*3946279|F|0–23:C>T−1024468|F|0–22:C>T*	752.4–756.5	14.0	15.4	−58.7
	YRM	96.0	*1269410|F|0–8:G>A−7353602|F|0–9:A>G*	728.9–729.8	13.9	12.6	−8.5

a *QTL that overlap in the one-log support confidence intervals were assigned the same symbol*.

b *LR16B and LR16B-A, MDS and AUDPC for leaf rust at El Batán in 2016; LR17B and LR167-A, MDS and AUDPC for leaf rust at El Batán in 2017; LR17O and LR17O-A, MDS and AUDPC for leaf rust in Ciudad Obregón during the 2016–2017 (LR17O) seasons; LR18O and LR18O-A, MDS and AUDPC for leaf rust in Ciudad Obregón during the 2017–2018 (LR2018O); LRM, mean MDS for all the leaf rust environments; YR17 and YR17-A, MDS and AUDPC for stripe rust at Toluca during the 2017; YR18 and YR18-A, MDS and AUDPC for stripe rust at Toluca during the 2018; YRM, mean MDS for all the stripe rust environments*.

c *Peak position in centi-Morgans from the first linked marker of the relevant linkage group*.

d *Logarithm of odds (LOD) score*.

e *Percentages of phenotypic variance explained by individual QTL*.

f *Additive effect of resistance allele*.

#### QTL Mapping for APR to Stripe Rust

Four QTL, *QYr.cim-1BS, QYr.cim-2AL, QYr.cim-4DL*, and *QYr.cim-7BL*, with the resistant allele from KU3067 were identified. *QYr.cim-1BS* was detected in YR17, YR17A, YR18-A, and YR-M and explained 12.9–20.5% of the phenotypic variance. *QYr.cim-2AL* was detected in all the stripe rust environments with PVE of 5.4–12.5%. *QYr.cim-4DL* was stably detected in all the stripe rust environments and explained 12.1–29.8% of the phenotypic variances. *QYr.cim-7BL* was identified in all the stripe rust environments with PVE 12.6–19.3% (Table 4).

#### Possible Pleiotropic Rust Resistance QTL

Among the QTL detected, two showed possible pleiotropic resistance for both rusts. The first one, *QLr.cim-4DL*/*QYr.cim-4DL*, proved to be *Lr67*/*Yr46* based on the closely linked marker *Tm4* (Moore et al., 2015) the marker was validated on the parent Ku3067, thus, confirming the presence of the gene *Lr67*. Another QTL, *QLr.cim-7BL*/*QYr.cim-7BL* located on 7BL also showed pleiotropic resistance. *QYr.cim-7BL* overlapped the *Yr* seedling resistance gene *YrKU* and showed all-stage resistance to stripe rust.

Overall, a total of six QTL for leaf rust and four QTL for stripe rust were identified in the population (Table 4; Figure 2). The total phenotypic variance explained by detected QTLs ranged from 55.1 to 83.9% across the environments for leaf rust and 44.4 to 71.6% for stripe rust, confirming their significant effect in reducing rust severity.

### Average Effects of Two Potentially Pleiotropic Rusts QTL and Additive Effects of Leaf Rust and Stripe Rust QTL

**Figure 3:**
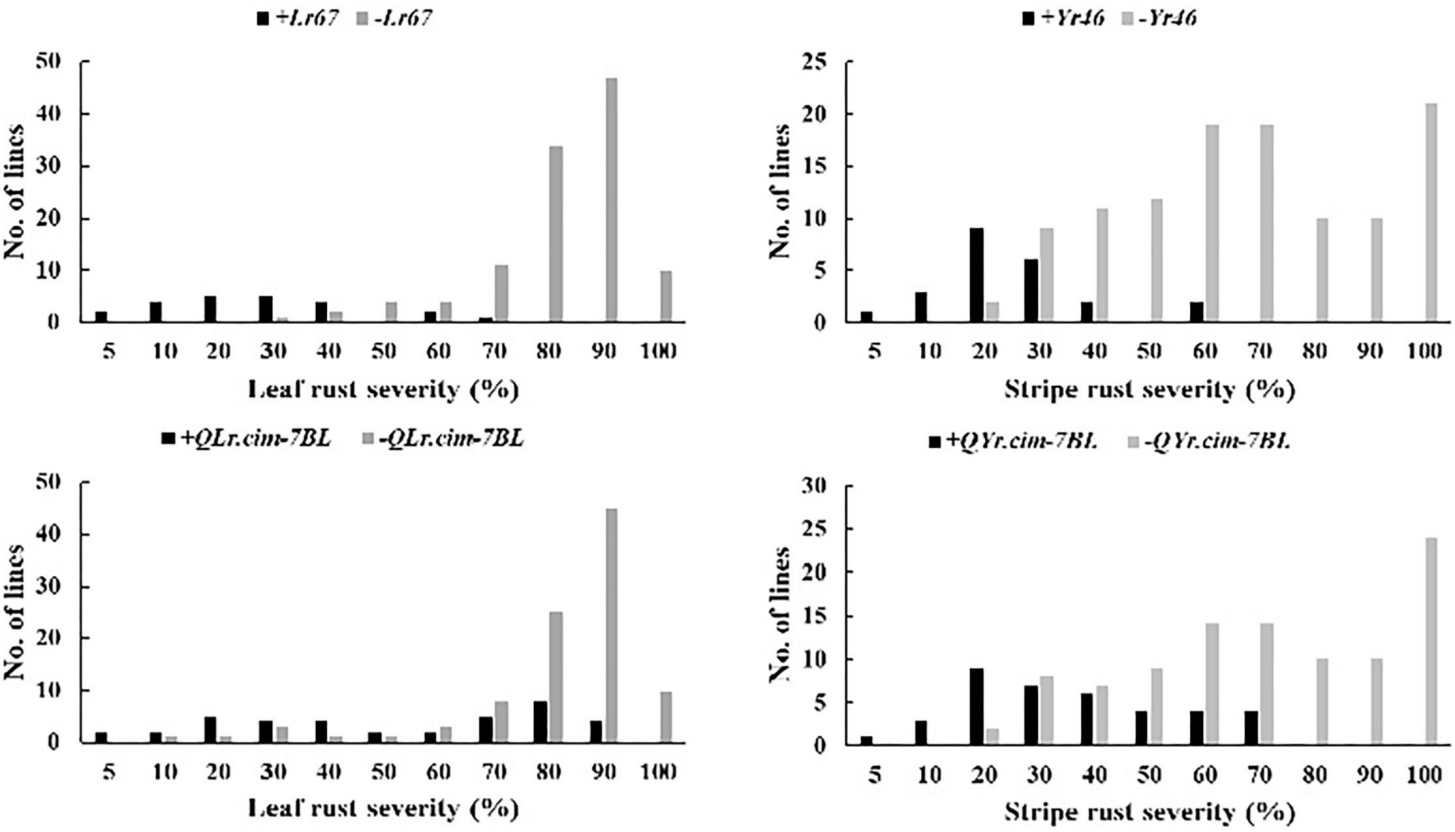
Comparison of KU3067/Apav BILs for mean MDS of LR and YR in the presence or absence of resistance allele for *Lr67/Yr46* and *QLr.cim-7BL/QYr.cim-7BL* in field trials.

Average effects of two potentially pleiotropic QTL were estimated in RIL carrying QTL and RIL lacking QTL were compared based on the closely linked flanking markers (Figure 3; Table 5). *Lr67*/*Yr46* showed significantly large effect in reducing leaf rust and stripe rust by 37.9–66.9 and 35.1–48.6%, respectively, in four leaf rust trials and two stripe rust trials (Table 5). Leaf rust severities in the presence of *Lr67* ranged from 4.3–60.1% compared with 21.2–100% in its absence. For *QLr.cim-7BL*/*QYr.cim-7BL*, there was a significant reduction in mean leaf rust and stripe rust severity by 20.0–35.0 and 29.8–40.9%, respectively. The mean leaf rust and stripe rust severities of RIL carrying the 7BL QTL, ranged from 3.4–90.0 and 5.3–65.0% compared with 7.5–100 and 17.5–100% in its absence, respectively (Figure 3).

**Table 5:** *T*-tests for the comparison of MDS for leaf rust and stripe rust in KU3067 × Apav BILs with and without the resistance alleles.

**QTL**	**No. of lines**	**LR2016B**	**LR2017B**	**LR2017O**	**LR2018O**	**LRM**	**YR2017**	**YR2018**	**YRM**
+*Lr67*/*Yr46*	23	18.7a	32.5a	19.5a	30.6a	25.3a	12.9a	32.5a	22.6a
–*Lr67*/*Yr46*	112	56.6b	76.1b	86.4b	91.9b	78.0b	61.5b	67.6b	64.2b
+*QLr.cim-7BL*/*QYr.cim-7BL*	37	36.4a	44.9a	50.6a	60.9a	48.1a	24.3a	40.3a	32.2a
–*QLr.cim-7BL*/*QYr.cim-7BL*	98	56.4b	78.3b	85.6b	89.9b	78.1b	65.2b	71.1b	68.2b

*Different letters within each column following the mean indicate significant differences based on the T-test (P < 0.01)*.

**Figure 4:**
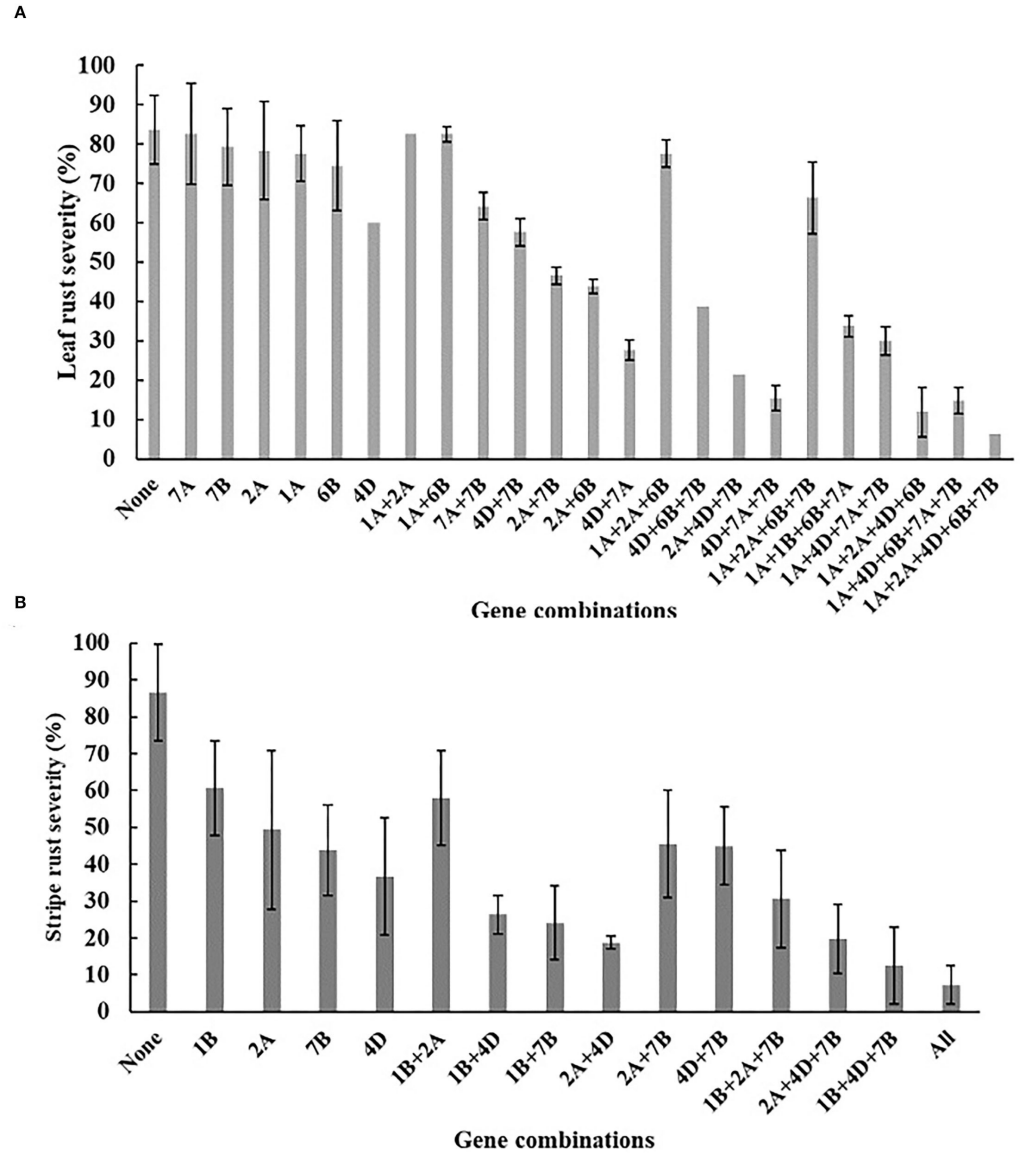
Mean MDS of lines carrying different combinations of QTLs. Lines containing the different QTL combinations were grouped together and the corresponding rust severities were averaged over environments. **(A)** Corresponds leaf rust combinations, **(B)** Corresponds to stripe rust evaluations. Means and standard errors of the means are shown.

The BILs were divided into 24 and 15 genotypes based on the presence of six-leaf rust and four stripe rust resistance QTL, and the additive nature of identified QTLs is shown in Figure 4. More QTLs imparted higher rust resistance in the BILs. For leaf rust, one line contained five QTL (4D + 7B + 1A + 2A + 6B) with mean MDS 6.3%. The mean MDS of the -QTL group reached 83.3%. For stripe rust, four lines with all four YR QTL had a mean MDS of 7.3%. In total, 46 RIL's without any of the reported stripe rust QTL had an MDS of 86.6%.

## Discussion

Genetic analyses indicated that four genes/loci controlled the APR resistance to leaf and stripe rusts in KU3067. In this mapping study, six QTL for leaf rust resistance and four for stripe rust resistance were identified using ICIM. The result of stripe rust was consistent with the estimated gene number, while there is a slight discrepancy with the genetic analyses for leaf rust. The Mendelian genetic approach for estimating gene numbers is based on the theoretical assumptions of polygenetic quantitative inheritance, where each gene is considered to contribute equally to the phenotype. However, it is common that variable effects of different QTL on phenotypes occur in the wheat genotypes, which results in segregating distortion of various phenotypic classes. Therefore, it is often observed that the estimated number of genes varies from the number of QTL identified. Yang et al. (2013) and Lan et al. (2015) also reported the discrepancy on the population Sujata/Avocet-*YrA* and Chapio/Avocet RIL populations, respectively. The estimated gene number usually represents the minimum number of polygenic loci segregating in a population, as is also evident in this study.

### *Lr67*/*Yr46* on 4DL

In this study, the most consistent QTL across all the environments was *Lr67*/*Yr46* on 4DL. *Lr67/Yr46* with a large effect explained 10.9–56.4 and 12.1–29.8% of the phenotypic variation for leaf and stripe rusts, respectively. The gene also confers resistance to stem rust and powdery mildew (Herrera-Foessel et al., 2014). *Lr67/Yr46* was originally transferred from PI250413, a Pakistani wheat accession, to Thatcher-derived line RL6077 (Dyck and Samborski, 1979). Earlier studies also confirm a larger phenotypic effect of *Lr67*/*Yr46* in Indian wheat lines Sujata (Lan et al., 2015) and New Pusa 876 (Ponce-Molina et al., 2018). This study is the first to report the presence of *Lr67*/*Yr46* in an Afghan landrace, which suggests a broad range of deployment of this gene in diverse wheat germplasm. This gene has been effective for over 60 years and remains a key gene in the development of lines with durable rust resistance in many breeding programs, including CIMMYT's program.

### QLr.cim-7BL/QYr.cim-7BL

*QLr.cim-7BL*/*QYr.cim-7BL* overlapped the *Yr* seedling resistance gene *YrKU*, indicating that *YrKU* showed all-stage resistance in the study. To date, six known *Yr* resistance genes *Yr39* (Lin and Chen, 2007), *Yr52* (Ren et al., 2012), *Yr59* (Zhou et al., 2014), *Yr67* (Li et al., 2009), *YrZH84* (Li et al., 2006), and *YrSuj* (Lan et al., 2015) as well as three leaf rust resistance genes *Lr14a, Lr14b* (McIntosh et al., 1995), and *Lr68* (Herrera-Foessel et al., 2012) have been reported on 7BL. The physical position of the *QLr.cim-7BL*/*QYr.cim-7BL* gene and closely linked markers is shown in Supplementary Table 2. *YrKU* (719.8–741.5 Mb) mapped at a similar position where *Yr52* (732.4 Mb), *Yr59* (723.9 Mb), *Yr67* (699.9–718.4 Mb), *YrSuj* (718.3–734.8 Mb), and *YrZH84* (718.3–723.9 Mb) are reported. As *Yr52* and *Yr59* are high-temperature adult-plant (HTAP) genes with susceptible infection type at the seedling stage, suggesting they are different from *YrKU*. *YrZH84*, a stripe rust seedling resistance gene, was identified in Chinese wheat cultivar “Zhou 8425B” however, does not show pleiotropic resistance to the leaf rust. *YrSuj* mapped in the Indian bread wheat cultivar Sujata (Lan et al., 2015) with the pleiotropic effect on APR to leaf rust is similar to our findings. Lan et al. (2015) also reported that *YrSuj* and *Yr67* from Indian variety C591 might be same (Lan et al., 2015). The relationship among *YrKU, Yr67*, and *YrSuj* will be confirmed through the allelism test in the next step to confirm if it is a known gene or a new gene at that locus.

For leaf rust, *Lr14a* and *Lr14b*, located on 7BL, are seedling resistance genes and seedling tests of the RIL population displayed infection type “4” or “3+,” indicating the two genes are ineffective against the *Pt* races that were tested, and hence, they are different from *QLr*.*cim*-*7BL*. *Lr68* is a slow-rusting resistance gene flanked by markers *cs7BLNLRR* and *Xgwm146* was mapped in CIMMYT line Arula (Herrera-Foessel et al., 2012). *QLr.cim-7BL* (729.4–753.8 Mb) mapped at a similar position with *Lr68* (752.2–752.8 Mb); but the absence of *Lr68* in KU3067 was confirmed by the linked molecular markers csGS and CAPS marker cs7BLNLRR (Herrera-Foessel et al., 2012). Hence *QLr.cim-7BL* may not be *Lr68*.

### QYr.cim-1BS

In total, six designated seedling *Yr* resistance genes, *Yr9* (Mettin et al., 1978), *Yr10* (Metzger and Silbaugh, 1970), *Yr15* (Gerechter-Amitai et al., 1989), *Yr24* (McIntosh and Lagudah, 2000), *Yr64* (Cheng et al., 2014), and *Yr65* (Cheng et al., 2014) were mapped on 1BS. *Yr10, Yr15, Yr24/Yr26, Yr64*, and *Yr65* were mapped at 5.5, 46.8–78.8, 218.9, 78.8, and 221.9–228.6 Mb, respectively, compared with *QYr.cim-1BS* at 3.7–7.0 Mb (Supplementary Table 2). In addition, the seedling tests showed that plants carrying *Yr10, Yr15*, and *Yr24*/*Yr26* were immune (IT 0) to *Pst* isolate Mex14.191, and plants with *Yr9* were susceptible (IT 8) while KU3067 showed IT 3, 4. Molecular markers of *Yr9, Yr10, Yr15*, and *Yr24*/*Yr26* also confirmed the absence of these genes in KU3067 (data not shown). Based to the chromosome position, seedling reaction and molecular markers detection, it can be concluded that *QYr.cim-1BS* is different from these genes and might be new.

### QYr.cim-2AL

Two known seedling resistance *Yr* genes, *Yr1* (Zadoks 1961), and *Yr32* (Eriksen et al., 2004), were mapped on 2AL. *Yr1* (Zadoks, 1961; Lupton and Macer, 1962) from winter wheat genotype Chinese 166 confers seedling resistance typically with IT 1 to pathotype Mex14.191, which is obviously different from IT 34 of KU3067. *Yr32* was originally characterized in cultivar Carstens V, and the seedling reaction of *Yr32* was unknown to *Pst* Mex14.191. *Yr32* was closely linked to *wmc198* at 711.5 Mb with genetic distance 2 cM compared with *QYr.cim-2AL* at 764.7–762.6 Mb. It can be concluded that *QYr.cim-2AL* is different from *Yr1* and *Yr32*. In addition, five QTL on 2AL for stripe rust resistance were reported in wheat varieties Récital (Dedryver et al., 2009), Camp Remy (Boukhatem et al., 2002) *Triticum monococcum* acc. pau14087 (Chhuneja et al., 2008), Solist (Christiansen et al., 2006), and Wasmo (Christiansen et al., 2006). *QYr.inra-2AL* and QYR2 from Récital and Camp Remy, respectively, were closely linked to *Xgwm382* at 774.3 Mb (Supplementary Table 2). QTL identified in Solist, Wasmo, and pau14087 were linked to *Xwmc170* at 715.3 Mb (Supplementary Table 2). In this study, *QYr.cim-2AL* at 764.7–762.6 Mb was 12 Mb away from *QYr.inra-2AL* and QYR2 and might be potentially new.

### QLr.cim-1AS

The known seedling resistance gene *Lr10* (Feuillet et al., 2003) and two QTL, *QLr.cim-1AS* from the Indian bread wheat cultivar Sujata (Lan et al., 2015) and *QLr.cau-1AS* from the American wheat cultivar Luke (Du et al., 2015), were mapped on 1AS. *Lr10* expressed IT 3 and 4 to *Pt* pathotypes MBJ/SP and MCJ/SP in the seedling test indicating it is ineffective against the tested *Pt*. *QLr.cim-1AS* (Lan et al., 2015) and *QLr.cau-1AS* (Du et al., 2015) were mapped at the distal end of 1AS with physical positions of 1.7–8.5 and 9.0 Mb (Supplementary Table 2). The physical position of KU3067 QTL *QLr.cim-1AS* (1.1–46.1 Mb) overlapped the latter two QTL (Supplementary Table 2). *QLr.cim-1AS* from Sujata showed pleiotropic resistance to stripe rust; however, we did not detect any effect of *QLr.cim-1AS* on stripe rust in this study. The relationship of these three QTL needs further analysis in the next step.

### QLr.cim-2AL

Leaf rust resistance gene *Lr38* introgressed from *Thinopyrym intermedium* to common wheat was mapped on 2AL (Friebe et al., 1992). There is no previous report suggesting that land race KU3067 might possibly carry *Lr38*. In addition, six QTL for leaf rust resistance, *QLr.cimmyt-2AL* in Avocet (Rosewarne et al., 2013), *QLr.sfr-2AL* in Forno (Schnurbusch et al., 2004), *QLr.ubo-2A* in Lloyd (Maccaferri et al., 2008), *QLr.hebau-2AL* in Chinese Spring (Zhang et al., 2017), *QLr.ifa-2AL* in Capo (Buerstmayr et al., 2014), and QTL-2AL in Opata 85 (Nelson et al., 1997), were mapped on 2AL. These QTL mapped in the different physical positions with the KU3067 QTL based on the position of closely linked markers (Supplementary Table 2), except for *QLr.cimmyt-2AL* in Avocet in an unknown position. It is currently not known if *QLr.cimmyt-2AL* is in the same region as the KU3067 QTL.

### QLr.cim-6BL

Four known QTL *viz*. *QLr.fcu-6BL* (Chu et al., 2009), *QLr.cimmyt-6BL.1* (Rosewarne et al., 2013), *QLr.cim-6BL* (Lan et al., 2017), and *QLr.cimmyt-6BL.2* (William et al., 2006) with resistance allele from synthetic hexaploid wheat TA4152-60, and CIMMYT bread wheats Pastor, Bairds, and Pavon 76, respectively, were mapped on 6BL. The Pastor QTL *QLr.cimmyt-6BL.1* (Rosewarne et al., 2013) and Pavon 76 QTL *QLr.cimmyt-6BL.2* (William et al., 2006) were effective against both leaf rust and stripe rust. However, no pleiotropic resistance was detected for the QTL identified in KU3067. *QLr.cim-6BL* in this study mapped at 495.2–576.7 Mb and is obviously different from 17.1 Mb for *QLr.fcu-6BL*, 706.7 Mb for *QLr.cim-6BL*, and 426.4 Mb for *QLr.cimmyt-6BL.2* (Supplementary Table 2). It can be concluded that *QLr.cim-6BL* from KU3067 might be potentially new.

### QLr.cim-7AL

Till date, only one known *Lr* gene, *Lr20* (Browder, 1972), was mapped at about 668.0 Mb on 7AL. *Lr20* was a seedling resistance gene and was susceptible to the two *Pt* pathotypes tested indicating it is ineffective. Two minor QTL, *viz*. *QLr.hwwg-7AL* (Lu et al., 2017) and *QLr.mma-7AL* (Tsilo et al., 2014), were mapped at 701.9 and 722.9 Mb on 7AL, respectively (Supplementary Table 2). *QLr.cim-7AL* was located at 699.0–701.5 Mb near to *QLr.hwwg-7AL*.

### Potential Application of QTL for Leaf Rust and Stripe Rust in Wheat Breeding

In this study, six QTL for leaf rust and four QTL for stripe rust were identified in the KU3067 RIL population. The leaf rust and stripe rust resistance of KU3067 had remained effective for more than 60 years. Despite their inferior agronomic traits (e.g., low grain yield, lodging), KU3067 and lines with multiple QTLs and combinations in the population can serve as a valuable source of resistance for common wheat improvement. The tightly linked SNP markers identified in this study can be converted to KASP assays (Semagn et al., 2014) for marker-assisted selection (MAS) and pyramiding of APR genes to improve leaf rust and stripe rust resistance in wheat-breeding programs.

## Data Availability Statement

The data used to present the findings reported in this study is available upon request through the corresponding author.

## Author Contributions

PZ, SB, and RPS contributed to the manuscript preparation, phenotyping, and QTL analysis. CL and JH-E contributed to population development. ZL contributed to experimental design and analysis. EL contributed to manuscript preparation and critical review. All authors contributed to the article and approved the submitted version.

## Funding

This work was supported by the Australian Grains Research and Development Corporation (GRDC) with funding to the Australian Cereal Rust Control Program (ACRCP) and Accelerating Genetic Gains in Maize and Wheat (AGG) project funded by Bill and Melinda Gates Foundation (BMGF), the UK's Foreign, Commonwealth and Development Office (FCDO), The United States Agency for International Development (USAID), The Foundation for Food and Agricultural Research (FFAR), and National Natural Science Foundation of China (32001538 and 32161143007) for supporting this research.

## Conflict of Interest

The authors declare that the research was conducted in the absence of any commercial or financial relationships that could be construed as a potential conflict of interest.

## Publisher's Note

All claims expressed in this article are solely those of the authors and do not necessarily represent those of their affiliated organizations, or those of the publisher, the editors and the reviewers. Any product that may be evaluated in this article, or claim that may be made by its manufacturer, is not guaranteed or endorsed by the publisher.

## References

[B1] Bhavani S. Hodson D. P. Huerta-Espino J. Randhawa M. S. Singh R. P. (2019). Progress in breeding for resistance to Ug99 and other races of the stem rust fungus in CIMMYT wheat germplasm. Front. Agric. Sci. Eng. 6, 210–224. 10.15302/J-FASE-2019268

[B2] Bhavani S. Singh P. K. Qureshi N. He X. Biswal A. K. Juliana P.. (2021). Globally important wheat diseases: status, challenges, breeding and genomic tools to enhance resistance durability. Genomic Des. Biot. Stress Resist. Cereal Crop 59–128. 10.1007/978-3-030-75879-0_2

[B3] Bjarko M. E. Line R. F. (1988). Heritability and number of genes controlling leaf rust resistance in four cultivars of wheat. Phytopathology 78, 457–461. 10.1094/Phyto-78-457

[B4] Boukhatem N. Baret P. V. Mingeot D. Jacquemin J. M. (2002). Quantitative trait loci for resistance against yellow rust in two wheat-derived recombinant inbred line populations. Theor. Appl. Genet. 104, 111–118. 10.1007/s001220200013 12579435

[B5] Browder L. E. (1972). Designation of two genes for resistance to *Puccinia recondita* in *Triticum aestivum*. Crop Sci. 12, 705–706. 10.2135/cropsci1972.0011183X001200050049x

[B6] Buerstmayr M. Matiasch L. Mascher F. Vida G. Ittu M. Robert O.. (2014). Mapping of quantitative adult plant field resistance to leaf rust and stripe rust in two European winter wheat populations reveals colocation of three QTL conferring resistance to both rust pathogens. Theor. Appl. Genet. 127, 2011–2028. 10.1007/s00122-014-2357-0 25112204 PMC4145209

[B7] Caldwell R. M. (1968). Breeding for general and/or specific plant disease resistance, in Proceedings of the 3rd International Wheat Genetics Symposium, eds Findlay K. W. Shepherd K. W. (Canberra, ACT: Australian Academy of Science), 263–272.

[B8] Chen X. M. (2005). Epidemiology and control of stripe rust [*Puccinia striiformis* f. sp. tritici] on wheat. Can. J. Plant Pathol. 27, 314–337. 10.1080/07060660509507230

[B9] Cheng P. Xu L. S. Wang M. N. See D. R. Chen X. M. (2014). Molecular mapping of genes *Yr64* and *Yr65* for stripe rust resistance in hexaploid derivatives of durum wheat accessions PI 331260 and PI 480016. Theor. Appl. Genet. 127, 2267–2277. 10.1007/s00122-014-2378-8 25142874

[B10] Chhuneja P. Kaur S. Garg T. Ghai M. Kaur S. Prashar M.. (2008). Mapping of adult plant stripe rust resistance genes in diploid A genome wheat species and their transfer to bread wheat. Theor. Appl. Genet. 116, 313–324. 10.1007/s00122-007-0668-0 17989954

[B11] Christiansen M. J. Feenstra B. Skovgaard I. M. Andersen S. B. (2006). Genetic analysis of resistance to yellow rust in hexaploid wheat using a mixture model for multiple crosses. Theor. Appl. Genet. 112, 581–591. 10.1007/s00122-005-0128-7 16395570

[B12] Chu C. G. Friesen T. L. Xu S. S. Faris J. D. Kolmer J. A. (2009). Identification of novel QTLs for seedling and adult plant leaf rust resistance in a wheat doubled haploid population. Theor. Appl. Genet. 119, 263–269. 10.1007/s00122-009-1035-0 19396420

[B13] Clavijo B. J. Venturini L. Schudoma C. Accinelli G. G. Kaithakottil G. Wright J.. (2017). An improved assembly and annotation of the allohexaploid wheat genome identifies complete families of agronomic genes and provides genomic evidence for chromosomal translocations. Genome Res. 27, 885–896. 10.1101/GR.217117.116/-/DC1 28420692 PMC5411782

[B14] Dedryver F. Paillard S. Mallard S. Robert O. Trottet M. Nègre S.. (2009). Characterization of genetic components involved in durable resistance to stripe rust in the bread wheat ’Renan'. Phytopatholgy 99, 968–973. 10.1094/PHYTO-99-8-0968 19594316

[B15] Du Z. Che M. Li G. Chen J. Quan W. Guo Y.. (2015). A QTL with major effect on reducing leaf rust severity on the short arm of chromosome 1A of wheat detected across different genetic backgrounds and diverse environments. Theor. Appl. Genet. 128, 1579–1594. 10.1007/s00122-015-2533-x 25982130

[B16] Dyck P. L. Samborski D. J. (1979). Adult plant resistance in PI250413, an introduction of common wheat. Can. J. Plant Sci. 59, 329–332. 10.4141/cjps79-053

[B17] Eriksen L. Afshari F. Christiansen M. J. McIntosh R. A. Jahoor A. Wellings C. R. (2004). *Yr32* for resistance to stripe (yellow) rust present in the wheat cultivar carstens V. Theor. Appl. Genet. 108, 567–575. 10.1007/s00122-003-1456-0 14523516

[B18] Feuillet C. Travella S. Stein N. Albar L. Nublat A. Keller B. (2003). Map-based isolation of the leaf rust disease resistance gene *Lr10* from the hexaploid wheat (*Triticum aestivum* L.) genome. Proc. Natl. Acad. Sci. U.S.A. 100, 15253–15258. 10.1073/pnas.2435133100 14645721 PMC299976

[B19] Flor H. H. (1942). Inheritance of pathogenicity in *Melampsora lini*. Phytopathology 32, 653–669. 10.1094/Phyto-32-653 40934406

[B20] Friebe B. Zeller F. J. Mukai Y. Forster B. P. Bartos P. McIntosh R. A. (1992). Characterization of rust-resistant wheat-*Agropyron intermedium* derivatives carrying resistance against leaf, stripe and stem rust by C-banding, in situ hybridization and isozyme analysis. Theor. Appl. Genet. 83, 775–782. 10.1007/BF00226697 24202753

[B21] Gerechter-Amitai Z. K. van Silfhout C. H. Grama A. Kleitman F. (1989). *Yr15*-a new gene for resistance to *Puccinia striiformis* in *Triticum dicoccoides* sel. G-25. Euphytica 43, 187–190. 10.1007/BF00037912

[B22] Herrera-Foessel S. A. Lagudah E. S. Huerta-Espino J. Hayden M. J. Bariana H. S. Singh D.. (2011). New slow-rusting leaf rust and stripe rust resistance genes Lr67 and Yr46 in wheat are pleiotropic or closely linked. Theor. Appl. Genet. 122, 239–249. 10.1007/s00122-010-1439-x 20848270

[B23] Herrera-Foessel S. A. Singh R. P. Huerta-Espino J. Rosewarne G. M. Periyannan S. K. Viccars L.. (2012). *Lr68*: a new gene conferring slow rusting resistance to leaf rust in wheat. Theor. Appl. Genet. 124, 1475–1486. 10.1007/s00122-012-1802-1 22297565

[B24] Herrera-Foessel S. A. Singh R. P. Lillemo M. Huerta-Espino J. Bhavani S. Singh S.. (2014). *Lr67*/*Yr46* confers adult plant resistance to stem rust and powdery mildew in wheat. Theor. Appl. Genet. 127, 781–789. 10.1007/s00122-013-2256-9 24408377

[B25] Huerta-Espino J. Singh R. Crespo-Herrera L. A. Villaseñor-Mir H. E. Rodriguez-Garcia M. F. Dreisigacker S. (2020). Adult plant slow rusting genes confer high levels of resistance to rusts in bread wheat cultivars from Mexico. Front. Plant Sci. 11, 824. 10.3389/fpls.2020.00824 32760411 PMC7371971

[B26] International Wheat Genome Sequencing Consortium (IWGSC) (2018). Shifting the limits in wheat research and breeding using a fully annotated reference genome. Science 361, eaar7191. 10.1126/science.aar7191 30115783

[B27] Johnson R. (1988). Durable resistance to yellow (stripe) rust in wheat and its implications in plant breeding, in Breeding Strategies for Resistance to the Rusts of Wheat, eds Simmonds N. W. Rajaram S. (Mexico: CIMMYT).

[B28] Knott D. R. (1989). The Wheat Rusts—Breeding for Resistance. Berlin: Springer-Verlag. 10.1007/978-3-642-83641-1

[B29] Kumar S. Bhardwaj S. C. Gangwar O. P. Sharma A. Qureshi N. Kumaran V. V.. (2021). Lr80: A new and widely effective source of leaf rust resistance of wheat for enhancing diversity of resistance among modern cultivars. Theor. Appl. Genet. 134, 849–858. 10.1007/s00122-020-03735-5 33388887

[B30] Lan C. Basnet B. R. Singh R. P.;, Huerta-Espino, J. Herrera-Foessel S. A.;, Ren, Y.. (2017). Genetic analysis and mapping of adult plant resistance loci to leaf rust in durum wheat cultivar bairds. Theor. Appl. Genet. 130, 609–619. 10.1007/s00122-016-2839-3 28004134

[B31] Lan C. Zhang Y. Herrera-Foessel S. A. Basnet B. R. Huerta-Espino J. Lagudah E. S.. (2015). Identification and characterization of pleiotropic and co-located resistance loci to leaf rust and stripe rust in bread wheat cultivar sujata. Theor. Appl. Genet. 128, 549–561. 10.1007/s00122-015-2454-8 25613742

[B32] Large E. C. (1954). Growth stages in cereals illustration of the feekes scale. Plant Pathol. 3, 128–129. 10.1111/j.1365-3059.1954.tb00716.x

[B33] Li H. H. Ye G. Y. Wang J. K. (2007). A modified algorithm for the improvement of composite interval mapping. Genetics 175, 361–374. 10.1534/genetics.106.066811 17110476 PMC1775001

[B34] Li J. Dundas I. Dong C. Li G. Trethowan R. Yang Z.. (2020). Identification and characterization of a new stripe rust resistance gene *Yr83* on rye chromosome 6R in wheat. Theor. Appl. Genet. 133, 1095–1107. 10.1007/s00122-020-03534-y 31955232

[B35] Li Y. Niu Y. C. Chen X. M. (2009). Mapping a stripe rust resistance gene *YrC591* in wheat variety C591 with SSR and AFLP markers. Theor. Appl. Genet. 118, 339–346. 10.1007/s00122-008-0903-3 18946654

[B36] Li Z. F. Zheng T. C. He Z. H. Li G. Q. Xu S. C. Li X. P.. (2006). Molecular tagging of stripe rust resistance gene *YrZH84* in Chinese wheat line Zhou 8425B. Theor. Appl. Genet. 112, 1098–1103. 10.1007/s00122-006-0211-8 16450183

[B37] Lin F. Chen X. M. (2007). Genetics and molecular mapping of genes for race-specific all-stage resistance and non-race-specific high-temperature adult-plant resistance to stripe rust in spring wheat cultivar alpowa. Theor. Appl. Genet. 114, 1277–1287. 10.1007/s00122-007-0518-0 17318493

[B38] Lu Y. Bowden R. L. Zhang G. Xu X. Fritz A. K. Bai G. (2017). Quantitative trait loci for slow-rusting resistance to leaf rust in doubled-haploid wheat population CI13227 x Lakin. Phytopathology 107, 1372–1380. 10.1094/PHYTO-09-16-0347-R 28589757

[B39] Lupton F. G. H. Macer R. C. F. (1962). Inheritance of resistance to yellow rust (*Puccinia glumarum*) in seven varieties of wheat. Br. Mycol. Soc. 45, 21–45. 10.1016/S0007-1536(62)80032-1

[B40] Maccaferri M. Mantovani P. Tuberosa R. DeAmbrogio E. Giuliani S. Demontis A.. (2008). A major QTL for durable leaf rust resistance widely exploited in durum wheat breeding programs maps on the distal region of chromosome arm 7BL. Theor. Appl. Genet. 117, 1225–1240. 10.1007/s00122-008-0857-5 18712342

[B41] McIntosh R. A. Lagudah E. S. (2000). Cytogenetic studies in wheat XVIII. Gene *Yr24* for resistance to stripe rust. Plant Breed. 119, 81–83. 10.1046/j.1439-0523.2000.00449.x

[B42] McIntosh R. A. Wellings C. R. Park R. F. (1995). Wheat Rusts: An Atlas of Resistance Genes. Melbourne, VIC: CSIRO Publishing. 10.1071/9780643101463

[B43] McNeal F. H. Konzak C. F. Smith E. P. Tate W. S. Russell T. S. (1971). A uniform system for recording and processing cereal research data. US Dept. Agric. Res. Serv. ARS 34, 34–121.

[B44] Mettin D. Bluthner W. D. Weinrich M. (1978). Studies on the nature and the possible origin of the spontaneously translocated 1B-1R chromosome in wheat. Wheat Inform. Serv. 47–48, 12–16.

[B45] Metzger R. J. Silbaugh B. A. (1970). Inheritance of resistance to stripe rust and its association with brown glume colour in *Triticum aestivum* L. PI 178383. Crop Sci. 10, 567–568. 10.2135/cropsci1970.0011183X001000050035x

[B46] Moore J. W. Herrera-Foessel S. Lan C. Schnippenkoetter W. Ayliffe M. Huerta-Espino J.. (2015). A recently evolved hexose transporter variant confers resistance to multiple pathogens in wheat. Nat. Genet. 47, 1494–1498. 10.1038/ng.3439 26551671

[B47] Nelson J. C. Singh R. P. Autrique J. E. Sorrells M. E. (1997). Mapping genes conferring and suppressing leaf rust resistance in wheat. Crop Sci. 37, 1928–1935. 10.2135/cropsci1997.0011183X003700060043x

[B48] Peterson R. F. Campbell A. B. Hannah A. E. (1948). A dia-grammatic scale for estimating rust intensity on leaves and stems of cereals. Can. J. Res. 26, 496–500. 10.1139/cjr48c-033

[B49] Pinto da Silva G. B. Zanella C. M. Martinelli J. A. Chaves M. S. Hiebert C. W. McCallum B. D.. (2018). Quantitative trait loci conferring leaf rust resistance in exaploidy wheat. Phytopathology 108, 1344–1354. 10.1094/PHYTO-06-18-0208-RVW 30211634

[B50] Ponce-Molina L. J. Huerta-Espino J. Singh R. P. Basnet B. R. Lagudah E. Aguilar-Rincón V. H.. (2018). Characterization of adult plant resistance to leaf rust and stripe rust in Indian wheat cultivar ’new pusa 876'. Crop Sci. 58, 630–638. 10.2135/cropsci2017.06.0396

[B51] Raman H. Raman R. Kilian A. Detering F. Carling J. Coombes N. (2014). Genome-wide delineation of natural variation for pod shatter resistance in *Brassica napus*. PloS ONE 9, e101673. 10.1371/journal.pone.0101673 25006804 PMC4090071

[B52] Randhawa M. S. Singh R. P. Dreisigacker S. Bhavani S. Huerta-Espino J. Rouse M. N.. (2018). Identification and validation of a common stem rust resistance locus in two bi-parental populations. Front. Plant Sci. 871, 1788. 10.3389/fpls.2018.01788 30555507 PMC6283910

[B53] Ren R. S. Wang M. N. Chen X. M. Zhang Z. J. (2012). Characterization and molecular mapping of *Yr52* for high-temperature adult-plant resistance to stripe rust in spring wheat germplasm PI 183527. Theor. Appl. Genet. 125, 847–857. 10.1007/s00122-012-1877-8 22562146

[B54] Roelfs A. P. Singh R. P. Saari E. E. (1992). Rust Diseases of Wheat: Concepts and Methods of Disease Management. Mexico: CIMMYT.

[B55] Rosewarne G. M. Herrera-Foessel S. A. Singh R. P. Huerta-Espino J. Lan C. X. He Z. H. (2013). Quantitative trait loci of stripe rust resistance in wheat. Theor. Appl. Genet. 126, 2427–2449. 10.1007/s00122-013-2159-9 23955314 PMC3782644

[B56] Sansaloni C. Franco J. Santos B. Percival-Alwyn L. Singh S. Petroli C.. (2020). Diversity analysis of 80,000 wheat accessions reveals consequences and opportunities of selection footprints. Nat. Commun. 2020 111 11, 4572. 10.1038/s41467-020-18404-w 32917907 PMC7486412

[B57] Schnurbusch T. Paillard S. Schori A. Messmer M. Schachermayr G. Winzeler M.. (2004). Dissection of quantitative and durable leaf rust resistance in Swiss winter wheat reveals a major resistance QTL in the *Lr34* chromosomal region. Theor. Appl. Genet. 108, 477–484. 10.1007/s00122-003-1444-4 14523520

[B58] Semagn K. Babu R. Hearne S. Olsen M. (2014). Single nucleotide polymorphism genotyping using Kompetitive Allele Specific PCR (KASP): overview of the technology and its application in crop improvement. Mol. Breeding 33, 1–14. 10.1007/s11032-013-9917-x

[B59] Sharp P. J. Kreis M. Shewry P. R. Gale M. D. (1988). Location of b-amylase sequence in wheat and its relatives. Theor. Appl. Genet. 75, 286–290.

[B60] Singh R. P. Herrera-Foessel S. A. Huerta-Espino J. Bariana H. Bansal U. McCallum B.. (2012). *Lr34*/*Yr18*/*Sr57*/*Pm38*/*Bdv1*/*Ltn1* confers slow rusting, adult plant resistance to *Puccinia graminis tritici*, in Proceedings of the 13th International Cereal Rusts and Powdery Mildews Conference, ed Chen W. Q. (Beijing).

[B61] Singh R. P. Herrera-Foessel S. A. Huerta-Espino J. Lan C. X. Basnet B. R. Bhavani S.. (2013). Pleiotropic gene *Lr46*/*Yr29*/*Pm39*/*Ltn2* confers slow rusting, adult plant resistance to wheat stem rust fungus, in Proceedings Borlaug Global Rust Initiative, 2013 Technical Workshop (New Delhi), 17.

[B62] Singh R. P. Rajaram S. (1992). Genetics of adult-plant resistance to leaf rust in ’frontana' and three CIMMYT wheats. Genome 35, 24–31.

[B63] Tanaka H. Morris C. Haruna M. Tsujimoto H. (2008). Prevalence of puroindoline alleles in wheat varieties from eastern Asia including the discovery of a new SNP in puroindoline b. Plant Genet. Resour. 6, 142–152. 10.1017/S1479262108993151

[B64] Tsilo T. J. Kolmer J. A. Anderson J. A. (2014). Molecular mapping and improvement of leaf rust resistance in wheat breeding lines. Phytopathology 104, 865–870. 10.1094/PHYTO-10-13-0276-R 24521485

[B65] Voorrips R. E. (2002). MapChart: software for the graphical presentation of linkage maps and QTLs. J. Hered. 93, 77–78. 10.1093/jhered/93.1.77 12011185

[B66] Wang M. Chen X. (2017). Stripe rust resistance, in Stripe Rust, eds Chen X. M. Kang Z. S. (Berlin: Springer), 353–558.

[B67] William H. M. Singh R. P. Huerta-Espino J. Palacios G. Suenaga K. (2006). Characterization of genetic loci conferring adult plant resistance to leaf rust and stripe rust in spring wheat. Genome 49, 977–990. 10.1139/g06-052 17036073

[B68] Yang E. N. Rosewarne G. M. Herrera-Foessel S. A. Huerta-Espino J. Tang Z. X. Sun C. F.. (2013). QTL analysis of the spring wheat “Chapio” identifies stable stripe rust resistance despite inter-continental genotype × environment interactions. Theor. Appl. Genet. 126, 1721–1732. 10.1007/s00122-013-2087-8 23558982

[B69] Zadoks J. C. (1961). Yellow rust on wheat. Studies on epidemiology and physiological specialization. J. Pl. Ziekten 67, 69–256.

[B70] Zhang P. Yin G. Zhou Y. Qi A. Gao F. Xia X.. (2017). QTL mapping of adult-plant resistance to leaf rust in the wheat cross Zhou 8425B/Chinese spring using high-density SNP markers. Front. Plant Sci. 8, 793. 10.3389/fpls.2017.00793 28559910 PMC5432574

[B71] Zhang R. Singh R. P. Lillemo M. He X. Randhawa M. S. Huerta-Espino J.. (2019). Two main stripe rust resistance genes identified in synthetic-derived wheat line Soru# 1. Phytopathology 109, 120–126. 10.1094/PHYTO-04-18-0141-R 30070970

[B72] Zhou X. L. Wang M. N. Chen X. M. Lu Y. Kang Z. S. Jing J. X. (2014). Identification of *Yr59* conferring high-temperature adult-plant resistance to stripe rust in wheat germplasm PI 178759. Theor. Appl. Genet. 127, 935–994. 10.1007/s00122-014-2269-z 24487945

