# Supplementary material for: Identification and Characterization of Resistance Loci to Wheat Leaf Rust and Stripe Rust in Afghan Landrace “KU3067”
Source: Front Plant Sci. 2022 Jun 28;13:894528. doi: 10.3389/fpls.2022.894528 (PMC9274257; doi:10.3389/fpls.2022.894528)
Supplement: Supplementary file 1 [file Data_Sheet_1.docx]

**Supplementary Table 1 Length and number of markers of linkage map in each chromosome constructed using IciMapping 4.1**

| Linkage map | Length (cM) | No. of markers | Marker density (cM/Marker) |
| --- | --- | --- | --- |
| 1A.1 | 103.0 | 143 | 0.72 |
| 1A.2 | 32.6 | 40 | 0.82 |
| 1A.3 | 26.9 | 31 | 0.87 |
| 2A.1 | 116.9 | 205 | 0.57 |
| 2A.2 | 55.3 | 72 | 0.77 |
| 3A.1 | 144.9 | 140 | 1.04 |
| 3A.2 | 13.8 | 36 | 0.38 |
| 3A.3 | 12.1 | 31 | 0.39 |
| 4A.1 | 39.8 | 105 | 0.38 |
| 4A.2 | 101.4 | 103 | 0.98 |
| 5A | 149.5 | 188 | 0.80 |
| 6A.1 | 44.6 | 93 | 0.48 |
| 6A.2 | 101.6 | 93 | 1.09 |
| 7A.1 | 110.5 | 142 | 0.78 |
| 7A.2 | 68.7 | 95 | 0.72 |
| 7A.3 | 68.9 | 99 | 0.70 |
| A genome | 1190.5 | 1616.0 | 0.74 |
| 1B.1 | 74.1 | 184 | 0.40 |
| 1B.2 | 82.5 | 80 | 1.03 |
| 2B.1 | 165.6 | 258 | 0.64 |
| 2B.2 | 25.9 | 34 | 0.76 |
| 3B.1 | 92.8 | 237 | 0.39 |
| 3B.2 | 7.0 | 45 | 0.16 |
| 4B.1 | 14.4 | 56 | 0.26 |
| 4B.2 | 4.5 | 15 | 0.30 |
| 5B.1 | 107.0 | 240 | 0.45 |
| 5B.2 | 62.7 | 105 | 0.60 |
| 6B.1 | 147.6 | 199 | 0.74 |
| 6B.2 | 23.8 | 60 | 0.40 |
| 7B | 122.8 | 196 | 0.63 |
| B genome | 930.7 | 1709.0 | 0.54 |
| 1D.1 | 93.6 | 81 | 1.16 |
| 1D.2 | 6.9 | 18 | 0.38 |
| 2D | 31.6 | 41 | 0.77 |
| 3D.1 | 85.5 | 109 | 0.78 |
| 3D.2 | 74.6 | 96 | 0.78 |
| 3D.3 | 16.2 | 40 | 0.41 |
| 4D | 21.5 | 46 | 0.47 |
| 5D | 251.5 | 79 | 3.18 |
| 6D.1 | 89.1 | 103 | 0.87 |
| 6D.2 | 16.6 | 30 | 0.55 |
| 7D.1 | 43.6 | 105 | 0.42 |
| 7D.2 | 12.0 | 21 | 0.57 |
| D genome | 742.7 | 769 | 0.97 |
| Total | 2863.9 | 4094 | 0.70 |

**Supplementary Table 2 Comparison of physical positions of the QTL identified in the present study with those reported previously**

| QTL | Marker interval | Physical map (Mb) | Origin | Reference |
| --- | --- | --- | --- | --- |
| *QYr.cim-1BS* | *4991863\|F\|0--7:C>A---1043386\|F\|0--14:A>G* | 3.7-7.0 | KU3067 |  |
| *Yr9* | *-* | - | Almus | Mettin et al. 1978 |
| *Yr10* | *Xpsp3000* | 5.5 | PI 178383 | Metzger and Silbaugh 1970 |
| *Yr15* | *barc8-gwm413* | 46.8-78.8 | G-25 | Gerechter-Amitai et al. 1989 |
| *Yr24* | *Xgwm498- Xbarc187* | 218.9-361.0 | K733 | McIntosh and Lagudah 2000 |
| *Yr26* | *Xgwm498- Xbarc187* | 361.0-218.9 | γ80-1 | Ma et al. 2001 |
| *Yr64* | *gwm413-gdm33* | 78.8 | PI 331260 | Cheng et al. 2014 |
| *Yr65* | *gwm11-gwm18* | 221.9-228.6 | PI 480016 | Cheng et al. 2014 |
| *QYr.cim-2AL* | *5370736\|F\|0--13:C>T---1269847\|F\|0--7:T>A* | 764.7-762.6 | KU3067 |  |
| *Yr1* | *Xstm673acag* | - | Chinese 166 | Zadoks 1961 |
| *Yr32* | *wmc198* | 711.5 | Carstens V | Eriksen et al. 2004 |
| *QYr.inra-2AL* | *Xgwm382* | 774.3 | Récital | Dedryver et al. 2009 |
| *QYR2* | *Xgwm382* | 774.3 | Camp Remy | Boukhatem et al. 2002 |
| *QYrtm.pau-2AX* | *Xwmc170* | 715.3 | *Triticum monococcum* acc. pau14087 | Chhuneja et al. 2008 |
| *2AL* | *wmc198-wmc170* | 711.5-715.3 | Solist | Christiansen et al. 2006 |
| *2AL* | *wmc198-wmc170* | 711.5-715.3 | Wasmo | Christiansen et al. 2006 |
| *Lr67/Yr46* | *2245206-1070439* | 234.4 | KU3067 |  |
| *QLr.cim-1AS* | *1240002\|F\|0--25:A>G---100055130\|F\|0--20:T>C* | 1.1-46.1 | KU3067 |  |
| *Lr10* | *Xsfr1* | - | ThatcherLr10 | Feuillet et al. 2003 |
| *QLr.cim-1AS* | *wPt-9752 / Xgdm33* | 1.7-8.5 | Sujata | Lan et al. 2015 |
| *QLr.cau-1AS* | *gpw2246* | 9.0 | Luke | Du et al. 2015 |
| *QLr.cim-2AL* | *1010332\|F\|0--55:G>A---3959842\|F\|0--17:A>G* | 644.4-654.3 | KU3067 |  |
| *QLr.cimmyt-2AL* | *wPT4419 - wPT8226* | - | Avocet | Rosewarne et al. 2012 |
| *QLr.sfr-2AL* | *cfa2263* | 430.4 | Forno | Schnurbusch et al. 2004 |
| *QLr.ubo-2A* | *wPT-386-310911* | distal region of 2AL | Lloyd | Maccaferri et al. 2008 |
| *QLr.hebau-2AL* | *Xwmc181 / BS00057060_51* | 732.6-733.2 | Chinese Spring | Zhang et al. 2017 |
| *QLr.ifa-2AL* | tPt-8937 WPT-8596 GWM312 | 712.8 | Capo | Buerstmayr et al. 2014 |
| *QTL-2AL* | *XksuE16 / Xbcd543* | 709.9/666.5 | Opata 85 | Nelson et al. 1997 |
| *QLr.cim-6BL* | *1200827\|F\|0--33:A>G---1001678\|F\|0--37:A>G* | 495.2-576.7 | KU3067 |  |
| *QLr.cim-6BL* | 277143 / 1234305(BARC134) | 706.7 | Bairds | Lan et al. 2017 |
| *QLr.fcu-6BL* | *Xbarc5–Xgwm469.2* | 17.1 | TA4152–60 | Chu et al. 2009 |
| *QLr.cimmyt-6BL.1* | *wPT6329–wPT5176* | - (pleiotripic to both diseases) | Pastor | Rosewarne et al. 2012 |
| *QLr.cimmyt-6BL.2* | *XpAGGmCGA1-gwm58* | 426.4 (pleiotripic to both diseases) | Pavon 76 | William et al. 2006 |
| *QLr.cim-7AL* | *1111941\|F\|0--50:T>C---4992965\|F\|0--22:A>G* | 699.0-701.5 | KU3067 |  |
| *Qlr.inra-7Aa (Lr20)* | *wPt6460, wPt3403, wPt5533, wPt0790 / gpw4050* | 668 | Balance | Azzimonti et al. 2014 |
| *QLr.hwwg-7AL* | *IWB42182 / IWB73053* | 701.9 | CI 13227 | Lu et al. 2017 |
| *QLr.mma-7AL* | *wPt1601,cfa2240* | 722.9 | MN98550-5 | Tsilo et al. 2014 |
| *QLr.cim-7BL* | *1269410\|F\|0--8:G>A-*--*3951774\|F\|0--29:G>C* | 729.4-753.8 | KU3067 |  |
| *Lr68* | *cs7BLNLRR-Xgwm146* | 752.2-752.8 | Parula | Herrera-Foessel et al. 2012 |
| *QYr.cim-7BL/YrKu* | *1269410\|F\|0--8:G>A-*--*3951774\|F\|0--29:G>C* | 729.4-753.8 | KU3067 |  |
| *Yr39* | *gwm131* | 609.2 | Alpowa | Lin and Chen 2007 |
| *Yr52* | *Xbarc182-Xwgp5258* | 743.8 | PI 183527 | Ren et al. 2012 |
| *Yr59* | *Xwgp5175 - Xbarc32* | 733.8 | PI 178759 | Zhou et al. 2014 |
| *Yr67* | *Xcfa2040-SC-P35M48* | 709.2-727.7 | C591 | Li et al. 2009 |
| *YrSuj* | *Xcfa2040-Xwmc526* | 727.7-746.9 | Sujata | Lan et al. 2015 |
| *YrZH84* | *Xcfa2040-Xbarc32* | 727.7-733.8 | Zhou 8425B | Li et al. 2016 |
| *QYr.caas-7BL.1* | *Xbarc176, XwPt-8106* | 561.2 | SHA3/ CBRD | Ren et al. 2012 |
| *Qhtap.wsu-7BL* | *Xwgp36,Xwgp45* | - | Alpowa | Lin and Chen 2007 |
| *QYrtb.orz-7BL* | *XwPt-2356* | 713.6 | Tubbs | Vazquez et al. 2015 |
| *QYr.caas-7BL.2* | *Xgwm577,XwPt-4300* | 720.6,715.1 | SHA3/ CBRD | Ren et al. 2012 |
| *QYr.cim-7BL* | *XwPt-3190,XwPt-1475* | 762.3 | Pastor | Rosewarne et al. 2012 |
| *QYr-7BL* | *Xwmc166* | 729.4 | Oligoculm | Suenaga et al. 2003 |
| *QYr-7BL* | *Xgwm146* | 752.8 | Strongfield | Singh et al. 2013 |
| *QYr.7BL* | *Xgwm344,XP32/M59* | 729.4 | Attila | Rosewarne et al. 2008 |

^a^ The physical positions of markers was available at IWGSC RefSeq v2.0 (https://urgi.versailles.inra.fr/jbrowseiwgsc/gmod_jbrowse/).

– means can not be identified in the IWGSC RefSeq v2.0.
